# Canine Parvovirus Asian Type 2 Variant C (CPV-2c) Detected in Côte d’Ivoire

**DOI:** 10.3390/v18060661

**Published:** 2026-06-11

**Authors:** Aristide A. Zobo, Vessaly Kallo, N’guessan F. Diobo, Comoé C. D. N’guessan, N’Cho P. Asseu, Lou S. A. Kouassi, Kouachi C. A. Asseu, Coulibaly T. R. Tiecoura, Geneviève L. Acapovi-Yao, Charles E. Lamien, William G. Dundon, Giovanni Franzo

**Affiliations:** 1 Laboratoire Central Vétérinaire de Bingerville (LCV), Abidjan, Côte d’Ivoire; aristidezobo@gmail.com (A.A.Z.); mmekoualaineekouassilou@gmail.com (L.S.A.K.); tiecoura_raoul@yahoo.fr (C.T.R.T.); 2 Direction des Services Vétérinaires (DSV), Abidjan, Côte d’Ivoire; vessalykallo@gmail.com; 3 UFR Biosciences, Université Félix Houphouët-Boigny, 22 BP 582, Abidjan 22, Côte d’Ivoire; diobonguessanfidele@yahoo.fr (N.F.D.); acapovigene@gmail.com (G.L.A.-Y.); 4 Direction Regionale, Ministère des Ressources Animales et Halieutiques (MIRAH), Bouafle, Côte d’Ivoire; nguessandess@gmail.com; 5 Cabinet Vétérinaires Zeudji d’Adzope, Adzope, Côte d’Ivoire; paneleasseu@gmail.com; 6 Société Nouvelle de Produits Vétérinaires en Côte d’Ivoire, 18 BP 2863, Abidjan 18, Côte d’Ivoire; asseukouachi@gmail.com; 7 Animal Production and Health Laboratory, Joint FAO/IAEA Centre of Nuclear Techniques in Food and Agriculture, Department of Nuclear Sciences and Applications, International Atomic Energy Agency, Wagramer Strasse 5, P.O. Box 100, A1400 Vienna, Austria; c.lamien@iaea.org; 8 Department of Animal Medicine, Production and Health, University of Padova, 35020 Legnaro, Italy; giovanni.franzo@unipd.it

**Keywords:** Canine parvovirus, Côte d’Ivoire, CPV-2, Vietnam, phylogenetic analysis, phylogeographic analysis

## Abstract

Canine parvovirus 2 (CPV-2) is a highly contagious virus transmitted among dogs through direct or indirect contact with infected feces. The disease poses a significant risk to unvaccinated animals, particularly young dogs, where mortality rates can be high. Globally, three CPV genotypes (e.g., 2a, 2b, and 2c) are known to circulate, with all three detected in several African countries. However, no cases of CPV-2 have been reported in Côte d’Ivoire until now. In this study, 12 fecal swabs collected from young dogs were positive for CPV-2 by conventional PCR. Subsequent sequencing and phylogenetic analysis of positive amplicons revealed that three samples belonged to the CPV-2c genotype, while the remaining nine showed high similarity to a CPV-2c variant recently reported in Asia, and more specifically, Vietnam. Phylogeographic analysis indicates multiple introduction pathways of the virus into Côte d’Ivoire. This study represents the first documented report of CPV-2 in Côte d’Ivoire and will be of interest to those working in the field of canine health.

## 1. Introduction

Canine parvovirus type 2 (CPV-2), one of the most clinically relevant infectious agents affecting dogs and cats [1], is classified within the species *Protoparvovirus carnivoran1* (family *Parvoviridae*, subfamily *Parvovirinae*), together with feline panleukopenia virus (FPV), which circulates among terrestrial felids and mustelids [2]. CPV-2 is a single-stranded DNA virus with a genome of approximately 5 kb, containing two open reading frames (ORFs). ORF1 encodes the non-structural proteins NS1 and NS2, whereas ORF2 encodes the structural proteins VP1 and VP2 through alternative splicing of the same mRNA. VP3 is derived from VP2 by proteolytic cleavage mediated by host enzymes [3]. Among these, VP2 and its corresponding genomic region are by far the most extensively studied, due to their pivotal role in host tropism and their relevance for the host immune response. Viral replication relies on host DNA polymerases and occurs in the nuclei of rapidly dividing cells, such as hematopoietic and intestinal epithelial cells in both young and adult animals, as well as in cardiomyocytes of fetuses and newborns. Clinically, CPV-2 infection is characterized by gastrointestinal disease, including anorexia, depression, vomiting, and mucoid or hemorrhagic diarrhea, often associated with leukopenia [3,4].

CPV-2, similarly to other ssDNA viruses, is characterized by a relatively high evolutionary rate. Following its emergence from feline panleukopenia virus through host-species adaptation, CPV-2 continued to evolve, leading to the replacement of the original CPV-2 strain by the so-called antigenic variants CPV-2a and CPV-2b (which also regained the ability to infect felines), and subsequently by CPV-2c [5,6,7,8]. These variants were initially defined based on distinctive amino acid profiles; however, discrepancies between phenotypic and genotypic classification have been reported, suggesting that several mutations may have arisen due to selective pressures and convergent evolution [9]. These variants rapidly achieved worldwide distribution.

The history of CPV-2 in Africa dates back several decades, with evidence of its presence reported in South Africa and Namibia in the late 1990s through serological investigations [10,11]. Subsequently, CPV-2 was reported in multiple African countries, including Egypt, Morocco, Nigeria, South Africa, Tanzania, Tunisia, and Zambia [12,13,14,15,16,17,18,19,20].

Most recently, a new “Asian CPV2c” variant has been described, which is characterized by distinct amino acids (aa) motifs in NS1 and VP2 [21,22,23]. Specifically, this variant is defined by the following aa substitutions: NS1 (60V, 544F, 545F, 630P), NS2 (60V, 151N, 152V), and VP2 (5A/G, 267Y, 297A, 324I, 370R). The Asian CPV-2c clade was initially identified in several Asian countries, where it has shown a progressive increase in prevalence, and has more recently been reported in multiple regions worldwide, including Africa [24,25,26,27]. Nevertheless, data on CPV-2 circulation in Africa, and on the distribution of the Asian CPV-2c variant in particular, remain limited.

A study in 2020 estimated the national population of dogs in households in Côte d’Ivoire to be around 1.4 M [28]. These dogs are used primarily as property and household protection against human intruders and wildlife, as well as for hunting. Although limited, companion-animal (pet) ownership is increasing in urban middle and upper-income households. Within this population, little is known about CPV-2. The present study contributes to filling this gap by reporting, for the first time, the identification, genetic characterization, and contextualization within the international scenario of an “Asian-like” CPV-2c strain in Côte d’Ivoire.

## 2. Materials and Methods

### 2.1. Sample Collection and Processing

Twelve fecal swabs were collected from young dogs (between 2 and 7 months) between January and September 2025 (Table 1). None of the dogs were vaccinated against CPV-2. The dogs (7 male, 5 female) had been brought to local veterinary clinics presenting symptoms which included diarrhea, vomiting, elevated temperature, and loss of appetite. The swab samples were sent to the Laboratoire Central Vétérinaire de Bingerville (LCV) for processing. DNA was extracted from the swabs using the QIAamp DNA minikit (Qiagen, Hilden, Germany). The DNA was screened by classical PCR according to Carrino et al. [29]. Briefly, a 1270bp segment of the VP2 gene was amplified using primers 1270F 5′-TGGAAATCACAGCAAACTC-3′ and 1270R 5′-AGTCTTGGTTTTAAGTCAGTATC-3. Thermal cycling conditions consisted of 95 °C for 15 min followed by 35 cycles of 95 °C for 30 s, 55 °C for 30 s, and 72 °C for 1 min, and a final elongation at 72 °C for 7 min. Positive amplicons were purified using a Wizard SV Gel and PCR Clean-up system kit (Promega, Madison, WI, USA) and sent for Sanger sequencing (Eurofins-Genomics, Köln, Germany). [Note: initial PCR screening was performed in a blinded manner (i.e., positive control was unavailable). The first positive amplicons were confirmed by sequencing and then used as positive controls for later screening].

### 2.2. Preliminary Strain Characterization

All available CPV VP2 sequences were downloaded from GenBank, selecting only those for which the collection country and date were available. Sequence preprocessing and filtering were conducted using a custom Python workflow based on Biopython (v1.84) [30]. The selected sequences were then aligned with the sequences from Côte D’Ivoire using MAFFT v7.526 [31] under the “add fragments” strategy, combined with “adjust direction accurately” to correct sequence orientation and “keep length” to preserve reference coordinates. Sequences were retained if they satisfied the following criteria: genetic distance ≤ 0.01 relative to at least one Côte d’Ivoire sequence, and gap fraction ≤ 0.05 relative to at least one Côte d’Ivoire sequence. To reduce redundancy while preserving spatiotemporal structure, sequences were grouped according to country and sampling year extracted from FASTA headers. Within each country–year group, sequences were ranked according to the number of informative nucleotide positions. The highest-ranking sequence was retained as the initial representative. Each subsequent sequence was compared iteratively against retained representatives within the same group. Sequences sharing ≥99.5% similarity (≤0.005 genetic distance) with any retained representative (country year pair) were classified as redundant and removed. A new sequence alignment was performed using MAFFT, and phylogenetic analysis was performed using IQ-TREE [32] under the maximum likelihood framework. The best-fitting nucleotide substitution model was selected according to the Bayesian Information Criterion (BIC) using the integrated ModelFinder algorithm (i.e., HKY + G). Branch support was assessed using the SH-like approximate likelihood ratio test (SH-aLRT) with 1000 replicates.

### 2.3. Phylogeographic Analysis

Phylogeographic analysis was performed on the VP2 region sequenced in the present study in order to maximize the phylogenetic signal. Based on the classification of the obtained strains within the Asian lineage of CPV-2c, as demonstrated in the previous step, the Côte d’Ivoire sequences were combined with a VP2 reference dataset derived from Franzo et al. (2023) [26], as well as with an updated African sequence dataset selected according to the above-mentioned sequence coverage criteria. In particular, key population parameters, including the evolutionary rate, time to the most recent common ancestor (tMRCA), and viral effective population size (Ne), were estimated using a serial coalescent approach implemented in BEAST X (v10.5.0) [33]. The nucleotide substitution model (i.e., HKY + G) was selected based on the Bayesian Information Criterion (BIC) calculated using jModelTest2 (v2.1.10) [34], while the molecular clock model (relaxed lognormal) and population dynamics (Bayesian Skygrid) [35,36] were chosen based on marginal likelihood estimation using stepping-stone and path-sampling approaches. A discrete state phylogeographic analysis was performed following the framework described by Lemey et al. [37], considering countries as discrete states. Bayesian stochastic search variable selection (BSSVS) was implemented to identify statistically supported migration rates. An asymmetric migration model was adopted to account for directionality in viral movements. To reduce dataset complexity and considering that African countries were the primary focus of the study, all non-African countries were grouped into macro-regions according to their geographical location.

Model parameters and trees were sampled every 5000 steps over a Markov chain Monte Carlo (MCMC) run of 50 million states, performed four times. Log and tree files were merged using the logcombiner utility of BEAST after removing the first 10% of the run as burn-in. Convergence and mixing were assessed through visual inspection using Tracer, and results were considered reliable only when effective sample size (ESS) values exceeded 200. A maximum clade credibility (MCC) tree was generated and annotated using TreeAnnotator (BEAST package). Migration rates were considered statistically supported when the Bayes factor (BF) was greater than 10, calculated using SpreaD3 (v0.9.7rc) [38]. Summary statistics and graphical representations were generated using R (v4.4.4) and associated libraries.

### 2.4. African-Focused Dataset

To better investigate the relationships between the Côte d’Ivoire sequences and those from other African countries, whose representation in the above-mentioned dataset was limited due to the availability of only partial VP2 sequences, an additional dataset was constructed. This dataset included African sequences and those clustering within the same clades as the Côte d’Ivoire sequences, as inferred from phylogenetic analysis. A genomic region of 430 bp was selected, representing the optimal compromise between maximizing geographic representativeness and retaining sufficient sequence length for robust inference.

## 3. Results

### 3.1. PCR Screening of Samples

All 12 samples were positive for CPV-2 by conventional PCR and produced a 1270bp amplicon, which was purified and sequenced.

### 3.2. Preliminary Sequence Characterization

Phylogenetic and phylogeographic analyses performed on the genomic region sequenced in the present study allowed the identification of a major clade that included nine of the sequences from Côte D’Ivoire (e.g., samples CIV/1/2025, CIV/2/2025, CIV/3/2025, CIV/4/2025, CIV/5/2025, CIV/6/2025, CIV/7/2025, CIV/24/2025, CIV/37/2025), clustering with Vietnamese sequences. For the remaining three sequences from Côte D’Ivoire (e.g., samples CIV/21/2025, CIV/23/2025, CIV/35/2025), clustering was less well resolved, with close relationships to sequences from samples identified from different geographic areas (Supplementary Figure S1). In both cases, the sequences grouped within the so-called Asian CPV-2 lineage and displayed the characteristic amino acid markers at key positions of the VP2 protein (i.e., 267Y, 297A, 324I, 370R).

### 3.3. Phylogeographic Analysis

**Figure 1:**
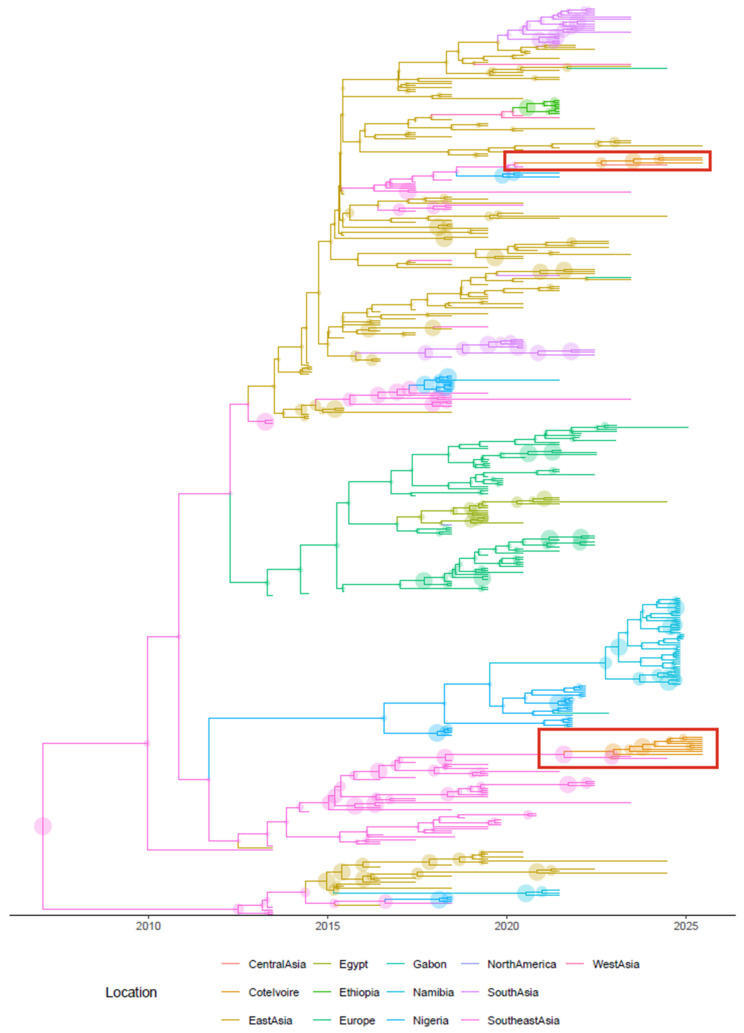
Time-scaled phylogenetic tree of the Asian CPV-2c lineage, including African sequences. Branches are colored according to the inferred geographic location, as indicated in the legend. Node sizes are proportional to posterior probabilities (non-African countries have been aggregated as macro-areas). The Côte D’Ivoire sequences have been highlighted with a red square.

The phylogeographic analysis, performed on the reference dataset of the Asian CPV-2c lineage, confirmed that the sequences from Côte D’Ivoire clustered into two main clades, comprising nine and three sequences, respectively. In both cases, Vietnam was inferred as the most likely origin, although an intermediate step through Kazakhstan may have occurred for the minor clade (Figure 1 and Supplementary Figure S2). The most recent common ancestor was estimated to date to around 2021 for the major clade and to 2022 for the smaller clade. However, in the latter case, a longer branch separated the Kazakhstan– Côte D’Ivoire group from the most likely Vietnamese ancestor, which was estimated to date back to 2019.

**Figure 2:**
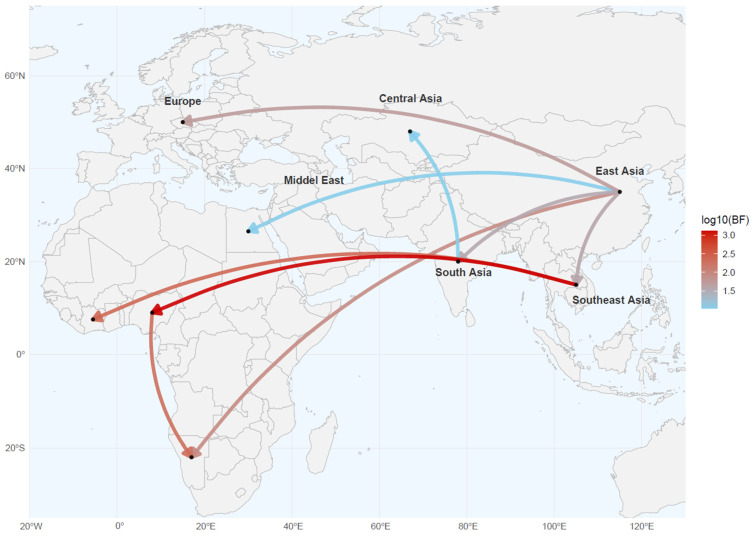
Map depicting statistically supported connections among countries and the directionality of migration rates [Support is color-coded as the Log10 Bayes factor (log10BF), maintaining only connections with BF > 10]. For graphical clarity and considering the specific aim of the study, non-African countries have been aggregated as macro-areas, and the regional centroid has been used as a representative.

Statistically supported migration pathways were identified across Africa, Asia, and Europe, with Asia, and particularly China (East Asia), appearing as the main source of viral spread. Statistically supported connections were identified between Southeast Asia and Côte d’Ivoire and Nigeria, as well as between East Asia and Egypt and Namibia. Additionally, connections between Nigeria and Namibia were observed (Figure 2).

### 3.4. Phylogenetic Analysis on the African Focused Dataset

**Figure 3:**
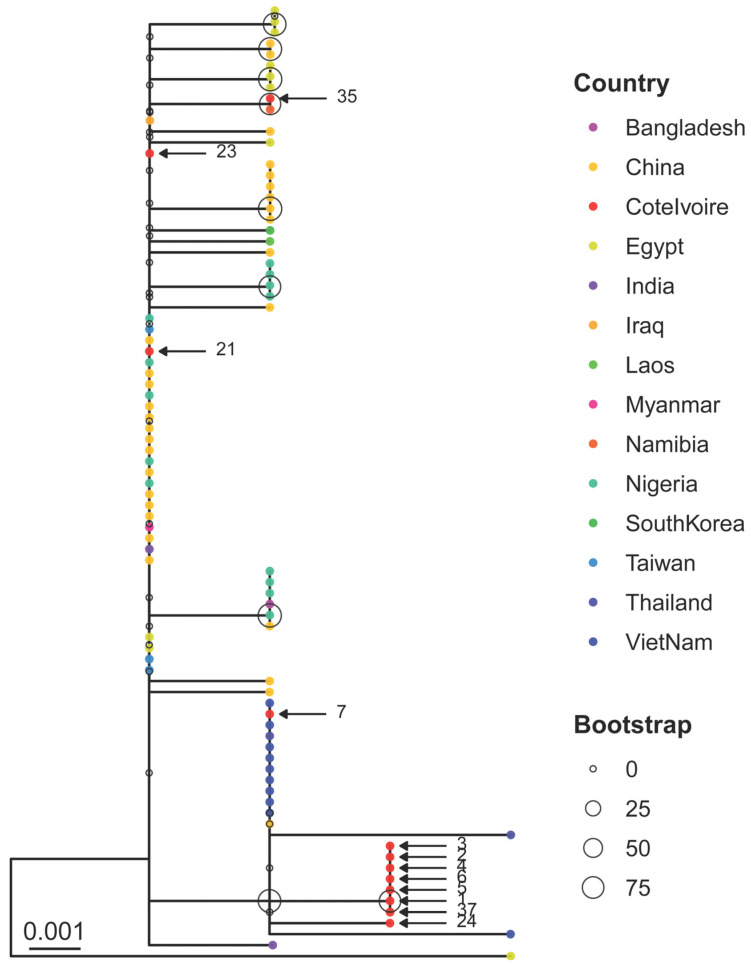
Midpoint-rooted phylogenetic tree based on the partial VP2 region (430bp), including sequences from Côte d’Ivoire and a broader panel of African and Asian countries. Tips are colored according to country of origin, while node support is indicated by bootstrap values. Côte d’Ivoire sequences are highlighted with arrows followed by the sample reference number (1, CIV/1/2025; 2, CIV/2/2025; 3, CIV/3/2025; 4, CIV/4/2025; 5, CIV/5/2025; 6, CIV/6/2025; 7, CIV/7/2025; 21, CIV/21/2025; 23, CIV/23/2025; 24, CIV/24/2025; 35, CIV/35/2025; 37, CIV/37/2025).

Phylogenetic analysis performed on the shorter VP2 region, which allowed for improved representation of African countries, confirmed the presence of two distinct clades. The major clade included the sequences from Côte D’Ivoire together with sequences of Asian origin, predominantly from China and Vietnam. In contrast, the inclusion of sequences from a broader geographical range revealed that strains belonging to the minor clade were closely related to those from several African countries, as well as from the Middle East (Iraq). Specifically, this clade included sequences from Egypt (North Africa), Namibia (Southern Africa), and Nigeria (West Africa). However, sequences from multiple Asian countries were also present within the same clade (Figure 3).

## 4. Discussion

Despite the extensive use of vaccines, CPV-2 still represents a major concern for companion animals as well as susceptible wildlife [39,40,41]. The epidemiology of the virus has been shaped by a combination of remarkable evolutionary potential and high transmission capability. Over time, new genetic and/or phenotypic variants have periodically emerged, some of which have achieved evolutionary success and global spread, with the “Asian” CPV-2c lineage representing one of the most recent examples [26].

African countries have traditionally been underrepresented in molecular epidemiological studies, largely due to limited resources and infrastructure required to perform such analyses, as well as cultural and socioeconomic factors that have historically assigned lower priority to the diagnosis and monitoring of infectious diseases in companion animals. However, this scenario is gradually changing and, although data remain limited, the increasing body of evidence is highlighting the growing relevance of CPV-2c variants in Africa, as reported in Namibia, Nigeria, and Zambia [42,43]. The evaluation of both amino acid profiles and phylogenetic analyses supports the classification of the viruses from Côte D’Ivoire into two main clades of the Asia-like CPV-2c lineage, consistent with independent introduction events. Phylogeographic reconstruction suggested that these two distinct introduction events occurred approximately in 2021–2022 and have been inferred to originate from Vietnam.

Understanding how canine viruses were introduced from Vietnam into Côte d’Ivoire remains challenging, given the currently limited data availability. Nevertheless, it is worth noting that Côte d’Ivoire is currently one of Vietnam’s main economic partners in Africa, and bilateral trade and exchanges have increased substantially in recent years [44].

In 2024, Vietnam exported about USD 413 million worth of goods to Côte d’Ivoire (e.g., rice, household goods, and industrial equipment) while Côte d’Ivoire exported about USD 831 million (e.g., cashew, rubber, and cotton) to Vietnam (https://oec.world/en/profile/bilateral-country/vnm/partner/civ (accessed on 22 April 2026)). Such connections may have facilitated viral introduction via the movement of people, such as business travelers or officials, potentially accompanied by their pets. However, in the absence of direct epidemiological evidence regarding animal movement, pet trade, or importation records, this hypothesis remains speculative. Illegal trade and uncontrolled animal movements could be another explanation that cannot be excluded. If confirmed, these hypotheses would support the implementation of stricter control measures on animal importation, including mandatory vaccination and/or testing.

The role of Asia as a major source of dissemination for the Asian CPV-2c lineage has been previously demonstrated [26], with epidemiological links identified between both North and West African countries [43]. These connections have also been supported by the present study. Although the overall pattern was confirmed, some differences compared with previous studies were also observed, including those reported by Franzo et al. 2022 [43], particularly regarding intra-African circulation patterns. These discrepancies can be explained by the specific aim of the present analysis, which focused on identifying the source of CPV-2 introduction into Côte d’Ivoire, as well as by differences in the reference dataset selection, conditioned by the sequenced genomic region and representing a compromise between sequence informativeness and country representativeness.

The present study was inevitably affected by the limited availability of African sequences and by sampling biases among different countries and time periods, which may have prevented the reconstruction of the complete viral transmission history and the identification of all epidemiological links. The long branches that were detected leading to the Côte d’Ivoire strains might in fact conceal other unsampled countries. Similarly, the limited number of strains that could be sequenced and analyzed might not be representative of the overall “within country” genetic variability, which could have originated from additional and alternative introduction events. Therefore, although the overall transmission pattern can be considered robust, the proposed introduction pathways and transmission mechanisms should be considered tentative and interpreted with caution, avoiding overinterpretation of the currently available data. Increased sequencing efforts and broader data-sharing initiatives across African countries would be highly beneficial to improving the reconstruction of viral epidemiology and evolutionary dynamics in the future.

Partial and non-standardized VP2 sequencing across laboratories represents a major limitation of this and other studies, hampering molecular epidemiological comparisons. Therefore, further efforts toward methodological harmonization are warranted. Nonetheless, it should be acknowledged that such standardization may be challenging in low-income settings, where replacing already validated diagnostic methods is not always considered a priority. Taking this into consideration, a dedicated phylogenetic analysis was performed in this study on a reduced genomic region, with the aim of increasing the inclusion of sequences from different countries, particularly from Africa, while retaining an adequate phylogenetic signal. However, this approach led to an inevitable reduction in sequence informativeness, leading to a lower resolution of the performed analyses and a reduced ability to discriminate minor differences among strains. The likely Asian origin of the most represented clade containing the sequences from Côte d’Ivoire was confirmed, as all these strains clustered within a lineage including sequences from China and, predominantly, Vietnam. In contrast, a less clearly defined geographic structure was observed for the remaining sequences, which grouped within a broader clade including, in addition to Asian sequences, several African ones (notably from Egypt, Nigeria, and Namibia). This pattern is more consistent with that reported by Franzo et al. 2022 [43], suggesting substantial within-Africa circulation following the initial introduction of the Asian lineage. The long branch observed in the phylogeographic reconstruction separating the Vietnamese ancestors from the sequences from Côte d’Ivoire may reflect unsampled or undetected intermediate migration events that likely occurred earlier during the spread of the Asian CPV-2c variants. The limited sequence length and the impossibility of including all available African sequences, due to incomplete overlap even within the selected region, still constrain the inferred connections among African countries. However, the combined evidence from previous studies and the present phylogenetic and phylogeographic analyses supports the existence of non-negligible within-continent viral dispersal. The movement of companion animals together with their owners likely represents the most plausible introduction pathway, especially considering the limited control of animal movements across many country borders in Africa.

The risk of CPV-2 transmission in Africa to wild canines, felines, and other susceptible species, such as hyenas, as documented in previous studies [45,46], must be carefully considered and should not be underestimated. Côte d’Ivoire hosts a limited wild canid community, primarily composed of side-striped jackals (*Lupulella adusta*), with the possible presence of small numbers of African wolves (*Canis lupaster*). In contrast, the country harbors a more diverse collection of wild felids, including the African golden cat (*Caracal aurata*), leopard (*Panthera pardus*), serval (*Leptailurus serval*), and caracal (*Caracal caracal*), as well as populations of spotted hyenas (*Crocuta crocuta*) [47,48]. Although the potential for CPV-2 transmission across the domestic–wildlife interface, and for subsequent regional or continental spread into endangered wildlife populations, remains insufficiently characterized, it clearly warrants further investigation.

Overall, the present study demonstrates the introduction of the recently emerged and rapidly expanding CPV-2c Asian lineage in Côte d’Ivoire. Multiple introduction pathways are likely, involving both direct importation from Vietnam, a country with which strong commercial relationships exist, and indirect introductions mediated by other African countries, where geographic proximity and porous borders may facilitate both viral importation and subsequent dissemination. Based on these findings, the need for more effective control strategies appears evident, including intensified screening and diagnostic activities. These results further highlight the importance of adopting standardized methodological approaches to enable robust comparisons of molecular epidemiological data across different studies and regions.

## Figures and Tables

**Table 1:** Canine samples tested in this study.

#	Name	Breed	Age (Months)	Sex	Collection Date	Location (City)	Clinical Outcome
1	CIV/21/2025	Poodle	6	F	14-01-2025	Bingerville	hospitalized-alive
2	CIV/23/2025	Landrace	2	M	03-02-2025	Bingerville	hospitalized-died
3	CIV/24/2025	Landrace	4	M	27-02-2025	Bingerville	died
4	CIV/35/2025	Laobé	2	M	16-05-2025	Adzope	hospitalized-alive
5	CIV/37/2025	Laobé	2	F	16-05-2025	Adzope	alive
6	CIV/1/2025	Landrace	4	M	08-2025	Bouaflé	died
7	CIV/2/2025	Landrace	4	M	08-2025	Bouaflé	alive
8	CIV/3/2025	Mixed	5	F	08-2025	Bouaflé	died
9	CIV/4/2025	Landrace	4	F	08-2025	Bouaflé	died
10	CIV/5/2025	Poodle	4	F	09-2025	Bouaflé	died
11	CIV/6/2025	Landrace	2	M	09-2025	Bouaflé	died
12	CIV/7/2025	Landrace	7	M	09-2025	Bouaflé	died

## Data Availability

The sequences have been deposited in GenBank under the following accession numbers: PX866683 to PX866694.

## Supplementary Materials

The following supporting information can be downloaded at: https://www.mdpi.com/article/10.3390/v18060661/s1, Figure S1: Midpoint-rooted Maximum likelihood phylogenetic tree inferred from the selected CPV-2 sequences. Figure S2, Time-scaled phylogenetic tree of the Asian CPV-2c lineage.

## Declarations

This document was prepared with the assistance of Microsoft M365 Copilot for language refinement and clarity. The authors retain full responsibility for the content.
