# Supplementary material for: Canine Parvovirus Asian Type 2 Variant C (CPV-2c) Detected in Côte d’Ivoire
Source: Viruses. 2026 Jun 11;18(6):661. doi: 10.3390/v18060661 (PMC13308472; doi:10.3390/v18060661)

Supplementary Figure S2. Time-scaled phylogenetic tree of the Asian CPV-2c lineage. Branches are coloured according to the inferred geographic location, as indicated in the legend. Node sizes are proportional to posterior probabilities (non-African countries have been aggregated as macro-areas).

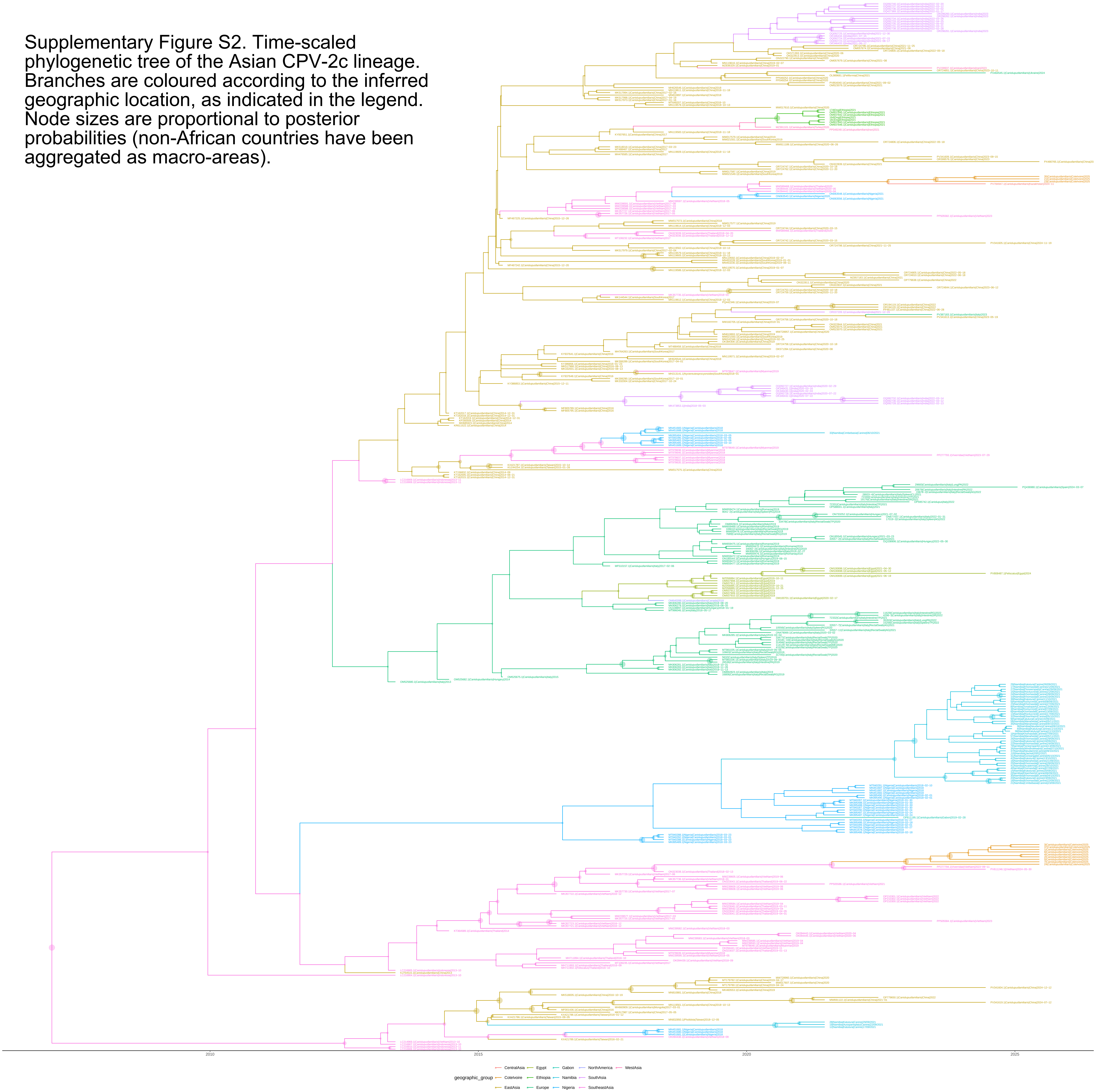

Supplement: Supplementary file 1 [file viruses-18-00661-s001.zip › Supplementary figure S2.pdf]
