# Supplementary figures and images for: Canine Parvovirus Asian Type 2 Variant C (CPV-2c) Detected in Côte d’Ivoire
Source: Viruses. 2026 Jun 11;18(6):661. doi: 10.3390/v18060661 (PMC13308472; doi:10.3390/v18060661)

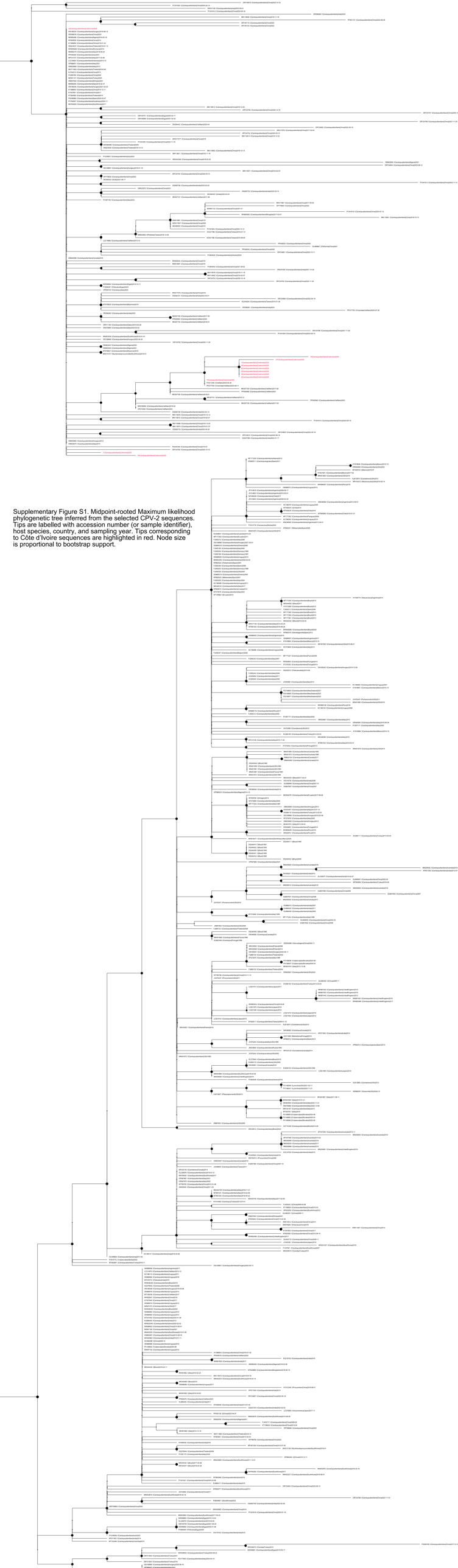

Supplement: Supplementary file 1 [file viruses-18-00661-s001.zip › Supplementary figure S1.pdf]
